# Genetic analysis of the GLUT10 glucose transporter (*SLC2A10*) polymorphisms in Caucasian American type 2 diabetes

**DOI:** 10.1186/1471-2350-6-42

**Published:** 2005-12-07

**Authors:** Jennifer L Bento, Donald W Bowden, Josyf C Mychaleckyj, Shohei Hirakawa, Stephen S Rich, Barry I Freedman, Fernando Segade

**Affiliations:** 1 Department of Biochemistry, Wake Forest University School of Medicine, Winston-Salem, North Carolina, 27157, USA; 2 Department of Internal Medicine, Wake Forest University School of Medicine, Winston-Salem, North Carolina, 27157, USA; 3 Department of Physiology and Pharmacology, Wake Forest University School of Medicine, Winston-Salem, North Carolina, 27157, USA; 4 Department of Public Health Sciences, Wake Forest University School of Medicine, Winston-Salem, North Carolina, 27157, USA; 5 Center for Human Genomics, Wake Forest University School of Medicine, Winston-Salem, North Carolina, 27157, USA

## Abstract

**Background:**

GLUT10 (gene symbol SLC2A10) is a facilitative glucose transporter within the type 2 diabetes (T2DM)-linked region on chromosome 20q12-13.1. Therefore, we evaluated GLUT10 as a positional candidate gene for T2DM in Caucasian Americans.

**Methods:**

Twenty SNPs including 4 coding, 10 intronic and 6 5' and 3' to the coding sequence were genotyped across a 100 kb region containing the SLC2A10 gene in DNAs from 300 T2DM cases and 310 controls using the Sequenom MassArray Genotyping System. Allelic association was evaluated, and linkage disequilibrium (LD) and haplotype structure of SLC2A10 were also determined to assess whether any specific haplotypes were associated with T2DM.

**Results:**

Of these variants, fifteen had heterozygosities greater than 0.80 and were analyzed further for association with T2DM. No evidence of significant association was observed for any variant with T2DM (all P ≥ 0.05), including Ala206Thr (rs2235491) which was previously reported to be associated with fasting insulin. Linkage disequilibrium analysis suggests that the SLC2A10 gene is contained in a single haplotype block of 14 kb. Haplotype association analysis with T2DM did not reveal any significant differences between haplotype frequencies in T2DM cases and controls.

**Conclusion:**

From our findings, we can conclude that sequence variants in or near GLUT10 are unlikely to contribute significantly to T2DM in Caucasian Americans.

## Background

Multiple genetic studies have been carried out that link human chromosome 20q13.1-13.2 to type 2 diabetes (T2DM) [1-5]. This linkage evidence has led investigators to search for T2DM susceptibility genes in this genomic region. Our laboratory has carried out analysis of specific genes [6-8] and developed high resolution physical maps of the region [9-11]. In an association analysis of genetic markers Price *et al*. [12] identified three regions of T2DM susceptibility. Among the genes mapped to the linkage disequilibrium regions, a novel facilitative glucose transporter (GLUT) was identified and designated GLUT10 (gene symbol SLC2A10) [6,13]. The gene spans 28 kb of genomic sequence, is split into 5 exons and 4 introns [6,13] and is expressed mainly in heart, liver, lung, skeletal muscle, pancreas, placenta, thyroid, and adipose tissue [6,13,14]. In Xenopus oocytes, human GLUT10 exhibited a high affinity, saturable 2-deoxy-D-glucose transport activity [6].

Glucose transport plays a central role in metabolism. Defects in glucose transport have been implicated in the reduced insulin sensitivity and in the increased insulin resistance observed in type 2 diabetes [14,15]. The genes coding for the classical GLUT1-5 proteins have been extensively analyzed for mutations contributing to type 2 diabetes, but no common causative mutation has been identified [16,17]. Both the function and genomic location of the novel SLC2A10 gene are consistent with a role in T2DM. In addition, Andersen *et al*. recently observed evidence for association of an Ala206Thr polymorphism in SLC2A10 with lower fasting insulin levels although they observed no evidence for association with T2DM [18]. We have carried out a systematic genetic evaluation of SLC2A10 to assess association with T2DM.

## Methods

### Subjects

The individual samples evaluated in this study consisted of a collection of 300 unrelated Caucasian T2DM patients with end-stage renal disease (ESRD), with a corresponding collection of 310 randomly-ascertained unrelated Caucasian subjects without known diabetes. Both cases and controls were recruited simultaneously. This group will be referred to as "T2DM-ESRD"; their ascertainment and recruitment have been previously described in detail [19]. The T2DM-ESRD subjects have a mean age at diagnosis of diabetes of 46.5 ± 12.8 years, mean BMI at recruitment of 28.5 ± 7.0, and mean maximum reported BMI of 36.1 ± 8.3, mean duration of diabetes > 15 years, and mean HbA1c of 8.6%.

### SNP selection and genotyping

Twenty SNPs used in this study were selected from the dbSNP public database (rs1004571, rs6012006, rs4810542, rs4810544, rs2425897, rs4428069, rs2425902, rs6094438, rs2235491, rs2076293, rs2425906, rs2425907, rs6094440, rs998422, rs2425911, rs3091904, rs6018008, rs707507, rs1059217, rs6090547). SNPs with frequency information were preferentially selected. The majority of the SNPs had minor allele frequencies greater than 0.2.

Genotyping was performed on a Sequenom MassArray Genotyping System using methods previously described [19]. Discordance between blind duplicate samples included in the genotyping was <0.9% and the call rate for each assay was set at ≥ 90%. Average call rate was 98.1% (range 95.6% to 100%).

### Statistical analysis

Pearson's test of homogeneity of proportions was applied to analyze allele frequency differences between diabetic and nondiabetic subjects SNPs were tested for Hardy-Weinberg equilibrium and pairwise linkage disequilibrium statistics D' calculated using the SNP-Analysis software package [20].

HAPLO.SCORE [21] was used to test for association of haplotypes within the case-control populations. HAPLO.SCORE uses posterior population-based frequency weighting of haplogenotypes that have prior consistency with observed heterozygous diplotypes. Variance estimates are inflated to account for uncertainty in specific haplotype assignment. We performed a global test of association between GLUT10 haplotypes and T2DM status, followed by individual tests of haplotype risk, in a collapsed 2 × 2 analysis. The skip.haplo option was set to 0.01. DANDELION [22], another expectation-maximization (EM) algorithm-based program for haplotype analyses was used to calculate haplotype frequencies in specific subject groups. A P < 0.05 was considered statistically significant.

## Results

**Figure 1:**
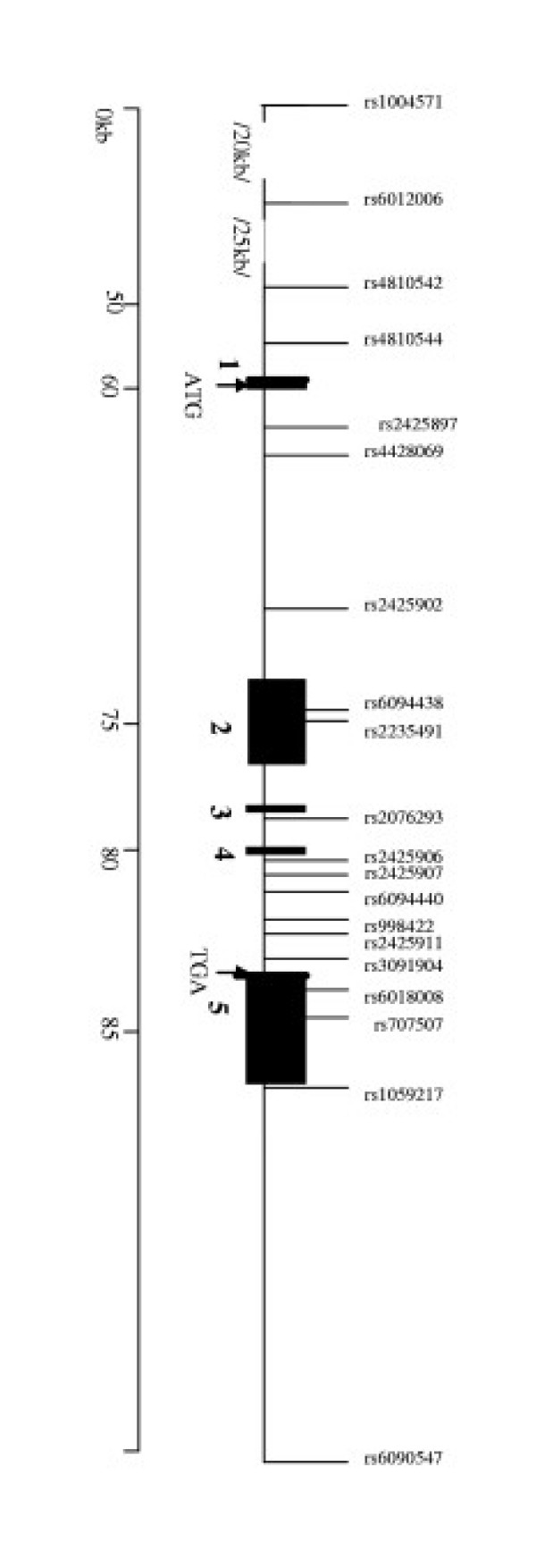
Genomic map of the SLC2A10 gene with the locations of the 20 genotyped SNPs. The shaded regions are exons, numbered 1–5. The ruler along the bottom represents the relative location and spacing of SNPs in kilobases within the 40 kb region containing SLC2A10. Note that this does not have a uniform scale.

To examine the association of SLC2A10 variants with T2DM, 20 SNPs spanning the genomic region were evaluated. The average SNP density was 1 SNP/5.2 kb with inter-SNP distances ranging from 0.078 kb to 26.856 kb. The location of the SNPs relative to the exon-intron structure of SLC2A10 is shown in Figure 1. The 20 SNPs were genotyped in a collection of Caucasian T2DM-ESRD cases (n = 300) and controls (n = 310) and single SNP association analysis was performed on those SNPs with minor allele frequencies greater than 0.20. Five of the SNPs are located in coding regions (rs6094438, rs2235491, rs6018008, rs707507, rs1059217), but only rs1059217 had a minor allele frequency greater than 0.20. The coding variant rs2235491 (Ala206Thr) had a low minor allele frequency of 0.04, but was included since it has been previously described as associated with reduced fasting insulin [18]. The remaining coding variants, rs6094438, rs6018008, rs707507, are so rare that they are very poorly informative with minor allele frequencies ≤ 0.01. In addition two SNPs in the noncoding regions, rs4428069 and rs609440, were also found to be nonpolymorphic.

In the control population, rs2076293 (P = <0.0001) and rs6090547 (P = 0.00043) did not conform to Hardy-Weinberg equilibrium. The allelic association analysis is summarized in Table 1, which shows the SNP identifier, the relevant alleles for each SNP, frequencies in cases and controls, and P-values. This analysis revealed moderate evidence of association between SNP rs2076293 and T2DM (P = 0.025). In addition, genotypic association analysis showed moderate evidence of association of this variant with T2DM under an additive model (P = 0.039). However, these results should be viewed with caution since the proportions of the genotypes for the variant are significantly out of Hardy-Weinberg equilibrium in the control population.

**Figure 2:**
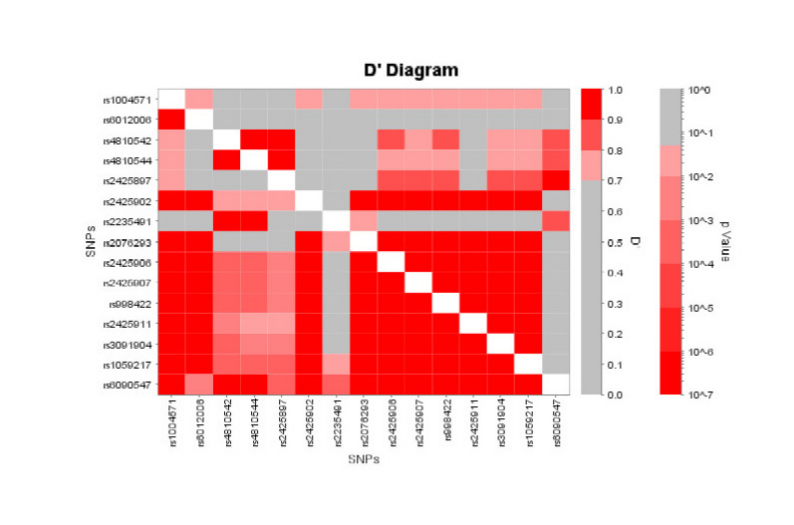
Marker-to-marker D' plot for the SLC2A10 SNPs. Inter-SNP D' values are graphically represented using LD Viewer which generates a color coded plot of the pair-wise statistics in the upper portion of the matrix, and a color coded plot of the calculated P-value for each LD measurement in the bottom portion of the matrix.

Linkage disequilibrium (LD) and haplotype structure of SLC2A10 were determined to assess whether any specific haplotypes were associated with T2DM. Inter-SNP D' statistics were calculated for the gene region as shown in Figure 2 using LD Viewer (unpublished). The upper portion of the matrix displays the calculated inter-SNP D' values, where a D' ≥ 0.70 is defined as high LD. The lower portion of the matrix shows the calculated P-values for each D' value, where a P-value ≤ 0.05 is significant (e.g. the calculated D' value is significant). Using these criteria (D' ≥ 0.70, P ≤ 0.05), SLC2A10 SNPs appear to be one LD block covering a 14 kb region which begins with variant rs2425902 in intron 1 and ends beyond the 3'-UTR with variant rs1059217.

Haplotype frequencies were estimated and association analyses performed using the programs Dandelion [22] and HAPLO.SCORE [21]. Shown in Table 2 are the results from the analyses evaluating 7 SNP haplotypes within the single major LD block of the SLC2A10 gene. Three common haplotypes are estimated and no significant evidence of haplotype association was observed with T2DM. It is important to note that although the variants rs2235491 and rs2235491 were within the LD block, they were removed from the analysis due to their low minor allele frequencies (rs2235491) or due to departure from Hardy-Weinberg equilibrium (rs2235491).

## Discussion

SNPs spanning the SLC2A10 genomic sequence have been genotyped in a Caucasian American T2DM case-control population. Most of the coding SNPs validated in this gene region were so rare in this study sample that they were uninformative for our analysis. The remaining SNPs did not show significant evidence for association to T2DM. This included SNP rs2235491 (Ala206Thr) which was previously reported to be associated with fasting insulin (but without evidence of associaton with T2DM) [18]. Linkage disequilibrium analysis suggests that there is one LD block covering a 14 kb region beginning with variant rs2425902 in intron 1 and ending with variant rs1059217 in the 3'UTR region. Haplotype analysis based on the LD structure did not reveal evidence for association with T2DM. However, the study is not powered (with the criterion MAF > 0.1) or designed to address less common SNPs or haplotypes. We have carried out a detailed power analysis for this case-control study group elsewhere (Bento et al, submitted). Briefly, assuming a T2DM disease prevalence of 10% and modest multiplicative genotype risk ratios of 1.5, the power to detect association at a nominal SNP significance level of 5% is 74.7% under an additive trend test with risk allele frequency of 10%, and >95% for risk allele frequency 20%.

## Conclusion

From these observations, we can conclude that variants in or near GLUT10 do not contribute substantially to T2DM in this sample of Caucasian Americans. This does not exclude the possibility that evidence for association could be observed in a larger case-control study or a study with cases ascertained in a different manner. However, another study has confirmed these negative association results. When examining SNPs in SLC2A10 using a Finnish population, no significant evidence for T2DM association with any SNP was observed [23]. In addition, this same study and another performed in a Danish population could not confirm the published association of Ala206Thr and fasting insulin [18, 24]. It is noteworthy that we have observed evidence for association to T2DM with the nearby PTPN1 gene, and to a lesser extent, with the HNF4A gene which is also located within 20q12-13.1.

## Abbreviations

SNP- single nucleotide polymorphism, T2DM- Type 2 diabetes, LD- linkage disequilibrium, ESRD- end stage renal disease, BMI- body mass index, GLUT10- glucose transporter 10, SLC2A10- (gene name for GLUT10) solute carrier family 2 member 10, PTPN1- protein tyrosine phosphatase non-receptor type 1, HNF4A- hepatocyte nuclear factor 4-alpha

## Competing interests

The author(s) declare that they have no competing interests.

## Authors' contributions

Dr. Bento carried out the majority of the genotyping and data analysis. The data analysis plan was designed and supervised by Drs. Mychaleckyj and Rich. Dr. Hirakawa carried out the initial laboratory studies identifying and genotyping SNPs in the coding sequence of GLUT10. Dr. Freedman oversaw recruitment, diagnosis, and clinical characterization of the subjects used in this study. Drs. Bowden and Segade conceived the overall study design and supervised the performance of the study. All authors participated in writing and editing the manuscript.

## Pre-publication history

The pre-publication history for this paper can be accessed here:



## Figures and Tables

**Table 1:** Association analysis for SLC2A10 SNPs in Caucasian T2DM-ESRD cases and controls.

		**Genotype Frequency: Cases**				**Genotype Frequency: Ccontrols**				
**SNP**	**Alleles**	**1/1**	**1/2**	**2/2**	**Frequency in Cases (n = 300)**	**1/1**	**1/2**	**2/2**	**Frequency in Controls (n = 310)**	**P-value**

rs1004571	A/G	25	125	146	0.30/0.70	28	139	141	0.32/0.68	0.426
rs6012006	A/C	26	104	159	0.27/0.73	29	130	135	0.32/0.68	0.063
rs4810542	G/A	3	40	246	0.08/0.92	0	33	271	0.05/0.95	0.094
rs4810544	C/T	4	33	257	0.07/0.93	0	33	277	0.05/0.95	0.263
rs2425897	A/T	0	17	279	0.03/0.97	0	19	287	0.03/0.97	0.78
rs2425902	G/A	25	119	151	0.29/0.71	37	120	146	0.32/0.68	0.207
rs2235491	A/G	4	16	276	0.04/0.96	1	16	288	0.03/0.97	0.37
*rs2076293	A/G	12	229	56	0.43/0.57	2	213	65	0.39/0.61	**0.025**
rs2425906	G/T	47	138	113	0.39/0.61	57	139	112	0.41/0.59	0.484
rs2425907	T/C	45	140	113	0.39/0.61	56	140	112	0.41/0.59	0.401
rs998422	G/A	46	139	114	0.39/0.61	58	138	108	0.42/0.58	0.289
rs2425911	G/C	26	123	146	0.30/0.70	40	121	144	0.33/0.67	0.213
rs3091904	C/T	44	147	107	0.39/0.61	59	139	111	0.42/0.58	0.541
rs1059217	C/T	46	137	104	0.40/0.60	60	137	111	0.42/0.58	0.567
*rs6090547	C/T	71	108	90	0.47/0.53	67	107	102	0.44/0.56	0.381

*Variant not consistent with Hardy-Weinberg proportions

P-values in bold indicates P ≤ 0.05

**Table 2:** Haplotype analysis using HAPLO.SCORE and Dandelion with 7 SNP (rs2425902, rs2425906, rs2425907, rs998422, rs2425911, rs3091904, rs1059217) SLC2A10 haplotypes.

**Haplotype**	**Case Frequency**	**Control Frequency**	**Hap-Score**	**Empirical Hap-specific P-value**	**Global simulated P-value**
ATCACTT	60.04%	58.23%	0.51	0.61	
GGTGGCC	29.15%	32.74%	-1.19	0.21	0.37
AGTGCCC	8.51%	8.22%	0.12	0.88	
